# Dr. Rachael Brooks Gleason

**Published:** 1905-04

**Authors:** 


### OBITUARY.

---

Dr. Rachael Brooks Gleason, formerly of Elmira, died in Buffalo, at the residence of her daughter, Dr. Adele A. Gleason, March 13, 1905, aged 84 years. Dr. Gleason had been engaged in medicine since 1851, and with her husband, Dr. Silas O. Gleason, spent the greater part of her active life in sanitarium work, having together established the Elmira Water Cure, now known as the Gleason Health Resort. This institution is now conducted by her son, Mr. Edward B. Gleason, with Dr. John C. Fisher as medical director.

